# Slow dissociation kinetics of fentanyls and nitazenes correlates with reduced sensitivity to naloxone reversal at the μ-opioid receptor

**DOI:** 10.1111/bph.17376

**Published:** 2024-10-22

**Authors:** Norah Alhosan, Damiana Cavallo, Marina Santiago, Eamonn Kelly, Graeme Henderson

**Affiliations:** 1 School of Physiology, Pharmacology and Neuroscience, University of Bristol, Bristol, UK; 2 College of Pharmacy, Princess Nourah bint Abdulrahman University, Riyadh, Saudi Arabia; 3 Macquarie Medical School, Faculty of Medicine Health and Human Sciences, Macquarie University, Sydney, New South Wales, Australia

**Keywords:** competitive antagonism, fentanyl, naloxone, nitazene, opioid, μ-opioid receptor

## Abstract

**Background and Purpose:**

Fentanyls and nitazenes are μ-opioid receptor agonists responsible for a large number of opioid overdose deaths. Here, we determined the potency, dissociation kinetics and antagonism by naloxone at the μ receptor of several fentanyl and nitazene analogues, compared to morphine and DAMGO.

**Experimental Approach:**

In vitro assays of G protein activation and signalling and arrestin recruitment were performed. AtT20 cells expressing μ receptors were loaded with a membrane potential dye and changes in fluorescence used to determine agonist potency, dissociation kinetics and susceptibility to antagonism by naloxone. BRET experiments were undertaken in HEK293T cells expressing μ receptors to assess Gi protein activation and β-arrestin 2 recruitment.

**Key Results:**

The apparent rate of agonist dissociation from the μ receptor varied: morphine, DAMGO, alfentanil and fentanyl dissociated rapidly, whereas isotonitazene, etonitazene, ohmefentanyl and carfentanil dissociated slowly. Slowly dissociating agonists were more resistant to antagonism by naloxone. For carfentanil, the slow apparent rate of dissociation was not because of G protein receptor kinase-mediated arrestin recruitment as its apparent rate of dissociation was not increased by inhibition of G protein-coupled receptor kinases (GRKs) with Compound 101. The in vitro relative potencies of fentanyls and nitazenes compared to morphine were much lower than that previously observed in in vivo experiments.

**Conclusions and Implications:**

With fentanyls and nitazenes that slowly dissociate from the μ receptor, antagonism by naloxone is pseudo-competitive. In overdoses involving fentanyls and nitazenes, higher doses of naloxone may be required for reversal than those normally used to reverse heroin overdose.

## 1 Introduction

### 1.1 Fentanyls and nitazenes

For the past 10 years, North America has been suffering a synthetic opioid drug epidemic that has resulted in hundreds of thousands of overdose deaths (Centers for Disease Control and Prevention, 2022; Public Health Agency of Canada, 2024). These opioid overdose deaths primarily involved fentanyls (fentanyl and its analogues). In the UK and mainland Europe, nitazenes, rather than fentanyls, are thought to pose a credible threat given the predicted decrease in heroin (diamorphine) availability from Afghanistan (Caulkins et al., 2024; Giraudon et al., 2024; Holland et al., 2024).

### 1.2 Naloxone antagonism

Whilst there are four main types of opioid receptor (μ, δ, κ and NOP receptors) in the nervous system, it is through the μ-opioid receptor that fentanyls and nitazenes produce their profound physiological effects, including respiratory depression, the main cause of death in overdose. Naloxone, the opioid antidote used to treat overdose, is generally regarded to be a competitive antagonist at the μ-opioid receptor (Corbett et al., 2006). For agonists and antagonists interacting at the same orthosteric binding site on a receptor, a competitive antagonist such as naloxone would be expected to reverse all agonists equally given that the antagonist binds to the unbound receptor and prevents agonist binding rather than physically displacing the agonist from the receptor (Ritter et al., 2024). However, there have been numerous reports of more naloxone, in the form of multiple or higher doses, being required to reverse overdoses involving fentanyls and nitazenes compared with heroin overdoses (Amaducci et al., 2023; Irvine et al., 2022; Moe et al., 2020). Reduced sensitivity to naloxone has also been observed in animal studies using sub-lethal doses of fentanyl (Elder et al., 2023; Hill et al., 2020).

### 1.3 Aims

In this study, we compared the potency and dissociation kinetics of a range of fentanyls (alfentanil, fentanyl, sufentanil, ohmefentanyl and carfentanil) and nitazenes (isotonitazene and etonitazene) at the μ-opioid receptor in vitro and compared them to the prototypic opioid agonists morphine and [D-Ala^2^, *N*-MePhe^4^, Gly-ol]-enkephalin (DAMGO). We also examined the ability of naloxone to antagonise the opioid agonists. Our results indicated that some fentanyls and nitazenes were less susceptible to naloxone antagonism than morphine and that this correlated with how slowly those agonists dissociated from the receptor.

## 2 Methods

### 2.1 Cell culture

‘Empty’ Flp-In modified AtT20 cells (AtT20FlpInWT) (ATCC CRL-1795; RRID:CVCL_4109) and Flp-In modified AtT20 cells recombinantly expressing the human μ-opioid receptor (AtT20FlpInMOR) were obtained from Prof Mark Connor, Macquarie University, Australia. The level of μ-opioid receptor expression in the AtT20FlpIn-MOR cells was 365 fmol mg^−1^ protein (O’Donnell, 2024). Cells were maintained in 75-cm flasks at 37°C and 5% CO_2_ in Dulbecco’s Modified Eagle’s Medium (DMEM) containing L-glutamine supplemented with 10% fetal bovine serum (FBS), 50 U ml^−1^ penicillin, 0.5 mg ml^−1^ streptomycin and 80 μg ml^−1^ hygromycin B. Human embryonic kidney 293T (HEK293T) cells were grown in 10-cm dishes in DMEM supplemented with 10% fetal bovine serum (FBS) and 50 U ml^−1^ penicillin and 0.5 mg ml^−1^ streptomycin and incubated in 5% CO_2_ at 37°C.

### 2.2 Membrane potential assay

The protocol used was a minor modification of that previously described (Knapman & Connor, 2014). When μ-opioid receptor-expressing AtT20 cells reached ≈ 90% confluency, they were detached by trypsinisation, resuspended in Leibovitz’s L-15 medium and plated into black, clear flat bottom 96-well assay plates coated with 0.01% poly-L-lysine solution. Each well received 90 μl of cell suspension and the plate was incubated overnight in air at 37°C. One hour prior to the experiment, cells were loaded with 90 μl of fluorescent blue membrane potential dye (supplied in the FLIPR Membrane Potential Assay Kit, Molecular Devices, San Jose, USA). Membrane potential dye and drug dilutions were prepared in a low potassium buffer (NaCl 145 mM, HEPES 22 mM, Na_2_HPO_4_ 0.338 mM, NaHCO_3_ 4.17 mM, KH_2_PO_4_ 0.441 mM, MgSO_4_ 0.407 mM, MgCl_2_ 0.493 mM, CaCl_2_ 1.26 mM, Glucose 5.56 mM, pH 7.4). Use of low potassium buffer reduced the extracellular potassium concentration in the solution bathing the cells during the experimental recordings from 5.6 to 2.9 mM, making the potassium equilibrium potential more negative, thus enhancing the amplitude of the opioid agonist-induced hyperpolarising responses. Fluorescence was measured from cells maintained at 37°C using a FlexStation 3 Multi-Mode Microplate Reader (Molecular Devices). Cells were excited at a wavelength of 530 nm and emission measured at 565 nm, with readings taken every 2 s. In control experiments, there was no decrease in the fluorescence measured in this way over a 500 s experimental run, as might have occurred with dye bleaching. Background fluorescence in wells with cells only (basal fluorescence RFU ≤ 10) or dye only (<10% of optimal basal fluorescence) was low and regarded as negligible.

Drug additions were performed using the robotic function of the FlexStation 3. All drugs were added in a volume of 10 μl and ejected at a speed of 16 μl s^−1^. The tip of the drug-containing pipette was placed at a height equivalent to the upper surface of the bathing fluid (180 μl for the first addition and 190 μl for the second). Following drug addition, the fluid in the well was ‘stirred’ by removing and re-injecting 10 μl of the bathing fluid three times as per the trituration setting of the FlexStation. In preliminary experiments examining the rate of potassium channel block by barium (a response that should have no intrinsic lag time), we estimated that the minimum response time measurable by the FlexStation following drug addition and mixing was 5.8 ± 0.4 s.

In the membrane potential assay experiments, we first examined the amplitude of the fluorescence signal from each well to ensure consistency of cell density, the condition of the cells and dye loading within and between experiments. All experiments were performed on wells that exhibited an initial fluorescence signal between 500 and 900 RFU. To control for differences in the basal level of the fluorescent signal between wells, the amplitude of subsequent drug-evoked responses was calculated as the percentage change from baseline, pre-drug addition, fluorescence readings in each well. Responses from wells that were injected with buffer alone rather than drug were subtracted to compensate for the small injection artefacts observed in some experiments. The change in the signal produced by the addition of buffer alone was ≤5% of the baseline.

### 2.3 Bioluminescence resonance energy transfer assays

To determine the relative ability of the opioid agonists to activate Gα_i_ G proteins and β-arrestin 2 translocation to the μ-opioid receptor, Bioluminescence Resonance Energy Transfer (BRET)^2^-based assays were used as described previously (Hill et al., 2018; Ramos-Gonzalez et al., 2023). For Gi activation, the assay monitored the separation of Gα_i1_ and Gγ_2_, whereas for arrestin translocation, the assay measured μ-opioid receptor and β-arrestin 2 association.

HEK293T cells were transiently transfected with the appropriate constructs when they reached 80% confluency in 100 mm dishes (for Gi activation – rat HA-μ-opioid receptor, Gα_i1_-*Renilla* luciferase II [RlucII] and GFP10-Gγ_2_; and for arrestin translocation – human μ-opioid receptor-RlucII and β-arrestin-2-GFP). The DNA (μg):Lipofectamine 2000 (μl) ratio was 1:2.7. Cells were then incubated for a further 48 h before the BRET assays were conducted. Immediately prior to each assay, cells were resuspended in clear DMEM and then transferred to a 96-well flat bottom white plate at 90 μl per well. Measurements of BRET were made at 37°C. Coelenterazine 400a, at a final concentration of 5 μM, was injected, and readings were taken 5 and 8 s later. BRET measurements were made on a CLARIOstar Omega plate reader (BMG LABTECH, Ortenberg, Germany) using 515 ± 30 nm (acceptor) and 410 ± 80 nm (donor) filters. BRET signals were determined as the ratio of the light emitted by acceptors (GFP10) over donor (RlucII). For Gi activation, BRET measurements were taken 2 min after agonist application, and for β-arrestin 2 association 10 min after agonist application. Agonist application resulted in a rapid decrease in the BRET signal between Gα_i1_-RlucII and GFP10-Gγ_2_ and an increase in the BRET signal between μ-opioid receptor-Rluc and β-arrestin-2-GFP. The ratio of the signal from the acceptor and donor was then calculated. Data were expressed either as percentage decrease for the Gi activation assay or as raw data with basal subtracted for the β-arrestin 2 recruitment assay.

### 2.4 Bias calculations

The International Union of Basic and Clinical Pharmacology (IUPHAR) guidelines for estimating G protein-coupled receptor (GPCR) ligand bias were followed to calculate bias (Kolb et al., 2022). Bias was calculated using two methods: using the operational model (ΔΔLog *τ*/*K*_A_) and the Log (*E*_max_/EC_50_) model. Concentration–response curves for each drug were generated in the G protein activation and β-arrestin 2 recruitment assays, and Log (*E*_max_/EC_50_) values were generated using three-parameter nonlinear regression fit. In each assay, the mean Log (*E*_max_/EC_50_) of each agonist was compared to the reference full agonist DAMGO to obtain Δ Log (*E*_max_/EC_50_). Then for each agonist, the Δ Log (*E*_max_/EC_50_) values between G protein activation and β-arrestin 2 recruitment assays were compared to acquire the ΔΔ Log (*E*_max_/EC_50_). For Log (*τ*/*K*_A_) analysis, agonist concentration–response curves for G protein activation and β-arrestin 2 recruitment were fitted to the Black–Leff operational model (Black & Leff, 1983) to generate Log (*τ*/*K*_A_) (transduction ratio) values (Kolb et al., 2022). A similar calculation to the one above for ΔΔ Log (*E*_max_/EC_50_) was undertaken to generate ΔΔLog (*τ*/*K*_A_).

### 2.5 Experimental design and data analysis

This manuscript complies with BJP’s recommendations and requirements on experimental design and analysis (Curtis et al., 2018; Curtis et al., 2022).

In the membrane potential and BRET assays, the order of addition of different drugs and their concentrations were randomised within individual experiments and across a series of experiments. Accordingly, the location of the basal or vehicle controls was different for each experiment. Blinding was not undertaken because of the complexity of the plate assays with multiple agonists and concentrations. Drug addition and data accumulation were automated by the software controlling the FlexStation and CLARIOstar 96-well plate readers. Raw data were downloaded into Excel spreadsheets for subsequent analysis. Membrane potential and BRET assays were conducted in duplicates. The mean of each duplicate was calculated and considered as *n* = 1. A priori sample size estimation indicated that a sample size of <5 would provide sufficient power for both membrane potential and BRET experiments. We therefore used a sample size of *n* = 5 in all experiments.

Data analysis, including statistical testing, was carried out in GraphPad Prism 9.0. For all assays, data points were excluded where their value was >3 × S.D. different from the mean of the other values. Concentration–response and naloxonereversal experiments with fentanyls and nitazenes were performed in two sets at different times. To allow comparison between each, we included morphine as a control in both. To construct concentration–response curves for each agonist, responses were normalised to that produced by morphine (1 μM) in the same set of experiments to remove any potential change in maximum response amplitude between the two sets of experiments.

pEC_50_, pIC_50_ and maximum response values were obtained by fitting concentration–response curves from each experiment individually by nonlinear regression (the initial value for no drug was constrained to zero or 100% as appropriate) and then combining values to give mean values ± standard error of the mean (SEM, *n* = 5). In the analysis of the concentration–response curves from the BRET experiments, it was not possible to fit the data for morphine, a weak partial agonist in the β-arrestin 2 translocation assay, without constraining the Hill slope to 1, and so for consistency, the Hill slope was constrained to 1 for all agonists.

Apparent dissociation half-times were obtained by fitting the decay of the agonist response after naloxone (10 μM) addition in each individual experiment to a custom equation downloadable to Graph-Pad from Pharmechanics. This took into account a variable ‘delay’ period following naloxone addition that was not different from the pre-drug baseline followed by the exponential decay to steady state. In each experiment, the goodness of fit was *r* ≥ 0.8. Note that, with regards to the rate of agonist dissociation from the receptors (see Figure 4 and Table 1), we use the term ‘apparent’ half time of agonist dissociation to indicate that although a high concentration of the antagonist naloxone has been added, the agonist has not been washed out, and so we cannot exclude that a small amount of agonist rebinding to the receptors may occur, thus slowing the rate of reversal.

All data were tested for normality using the D’Agostino and Pearson test and graphically using *Q*–*Q* plot and bar graphs. To test for statistical differences among pEC_50_, *E*_max_, pIC_50_ and apparent dissociation half-time values, all ligands were compared to morphine (membrane potential assay) or DAMGO (BRET assay) as the reference agonist using one-way analysis of variance (ANOVA). Post hoc Dunnett’s multiple comparisons were conducted only if the results showed a statistical significance (*F*-value achieved a *P*-value of < 0.05) and no significant variance inhomogeneity. For the correlation analyses, the nonparametric Spearman test was used as the data were not normally distributed and a two-tailed *P*-value of <0.05 taken to indicate significance.

### 2.6 Materials

The drugs used were alfentanil hydrochloride (Cayman Chemicals, Ann Arbor, USA), carfentanil and ohmefentanyl (Toronto Research Chemicals, Toronto, Canada), Compound 101 (Hello Bio, Bristol, UK), DAMGO (Bachem, St Helens, UK and Sigma–Aldrich, Dorset, UK), etonitazene hydrochloride fentanyl citrate, naloxone hydrochloride, U69593 (Sigma–Aldrich), isotonitazene (Cayman Chemicals), morphine hydrochloride (Macfarlan Smith, Edinburgh, UK), nociceptin (orpahnin FQ; Biotechne, Abingdon UK) and SNC80 (Tocris Bioscience, Bristol, UK). Drugs were made up as stocks in deionised water or dimethylsulfoxide (DMSO) and subsequently diluted in the appropriate experimental buffer. Drugs were made up in water except isotonitazene, etonitazene and Compound 101 which were initially dissolved in 100% DMSO and subsequently diluted in experimental buffer. The highest final concentration of DMSO used in each assay never exceeded 0.01%. The effect of 0.01% DMSO alone was not different to saline controls.

### 2.7 Nomenclature of targets and ligands

Key protein targets and ligands in this article are hyperlinked to corresponding entries in the IUPHAR/BPS Guide to PHARMACOLOGY http://www.guidetopharmacology.org and are permanently archived in the Concise Guide to PHARMACOLOGY 2023/23 (Alexander, Christopoulos, Davenport, Kelly, Mathie, Peters, Veale, Armstrong, Faccenda, Harding, Davies, et al., 2023; Alexander, Fabbro, Kelly, Mathie, Peters, Veale, Armstrong, Faccenda, Harding, Davies, Amarosi, et al., 2023; Alexander, Fabbro, Kelly, Mathie, Peters, Veale, Armstrong, Faccenda, Harding, Davies, Annett, et al., 2023; Alexander, Mathie, Peters, Veale, Striessnig, Kelly, Armstrong, Faccenda, Harding, Davies, Aldrich, et al., 2023).

## 3 Results

### 3.1 Opioid agonist concentration–response relationships in the AtT20 cell membrane potential assay

**Figure 1:**
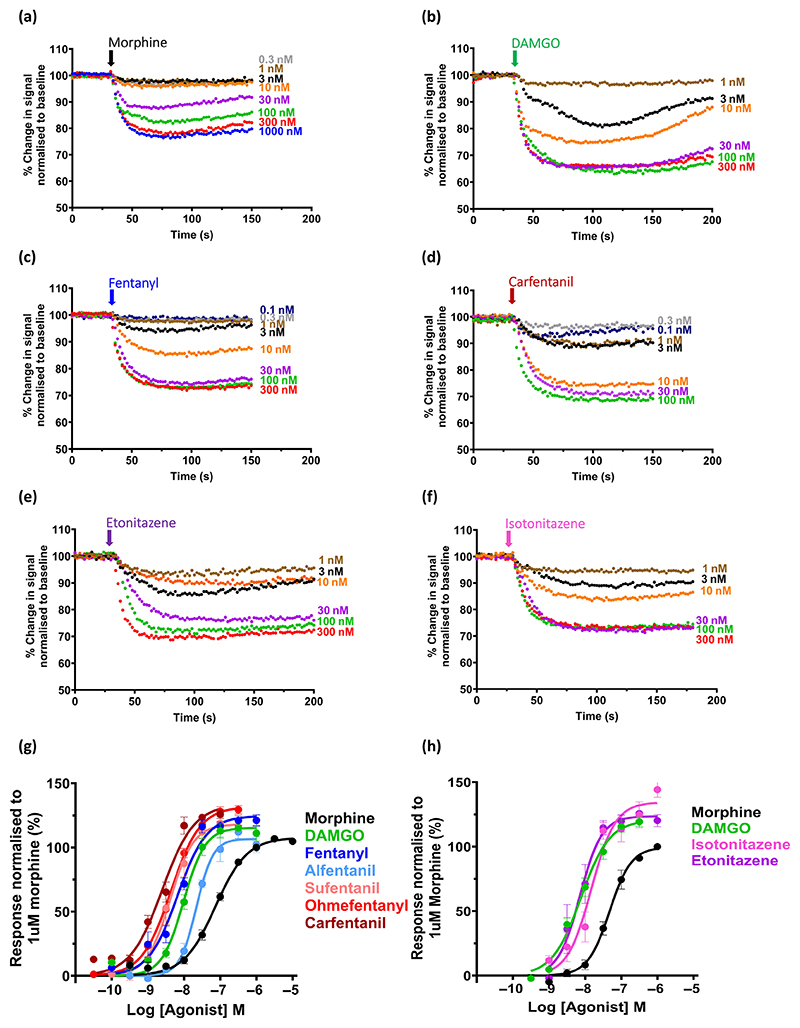
Concentration–response relationships for opioid-induced hyperpolarisation of μ-opioid receptor-expressing AtT20 cells. (a–f) Typical experimental traces for the change in membrane potential dye fluorescence signal produced by increasing concentrations of six opioid agonists—morphine, DAMGO, fentanyl, carfentanil, etonitazene and isotonitazene. Fluorescence values have been normalised to the pre-drug baseline. For each agonist, the data are from the same experiment and are typical of responses obtained in five experiments for each agonist. (g and h) Log concentration–response curves for the decrease in fluorescence induced by all the opioid agonists tested. Data for DAMGO, fentanyl, alfentanil, sufentanil, ohmefentanyl and carfentanil were obtained in a different set of experiments from those for etonitazene and isotonitazene. To facilitate comparison of all opioid agonists, morphine and DAMGO were included in both sets of experiments and changes in fluorescence normalised to the response induced by 1 μM morphine. Data shown are means ± SEM, *n* = 5 for each drug. Log concentration–response curves were constructed using nonlinear regression with the bottom of the curve constrained to zero. Calculated values of agonist pEC_50_, maximum response relative to morphine, Hill slope and relative potency to morphine are given in Tables 1 and 3.

The opioid agonists fentanyl, alfentanil, sufentanil, ohmefentanyl, carfentanil, etonitazene and isotonitazene, as well as the prototypic μ-opioid receptor agonists DAMGO and morphine, each produced a concentration-dependent decrease in fluorescence in μ-opioid receptor-expressing AtT20 cells loaded with membrane potential dye indicative of membrane hyperpolarisation following G protein-mediated G protein-coupled inwardly rectifying potassium channel (GIRK) activation (Figure 1). The rank order of potency was carfentanil ≥ sufentanil ≥ ohmefentanyl > fentanyl = etonitazene ≥ DAMGO > isotonitazene ≥ alfentanil > morphine (see Table 1 for pEC_50_ values). In this assay, the relative potencies of the fentanyls and nitazenes compared to morphine were lower than might be expected from in vivo antinociception experiments (Suzuki & El-Haddad, 2017; Hasegawa et al., 2022). Fentanyl, isotonitazene, ohmefentanyl and carfentanil evoked a higher maximum response than morphine (Figure 1g,h and Table 1).

### 3.2 Antagonism of opioid agonists by naloxone

#### 3.2.1 Reversal

**Figure 2:**
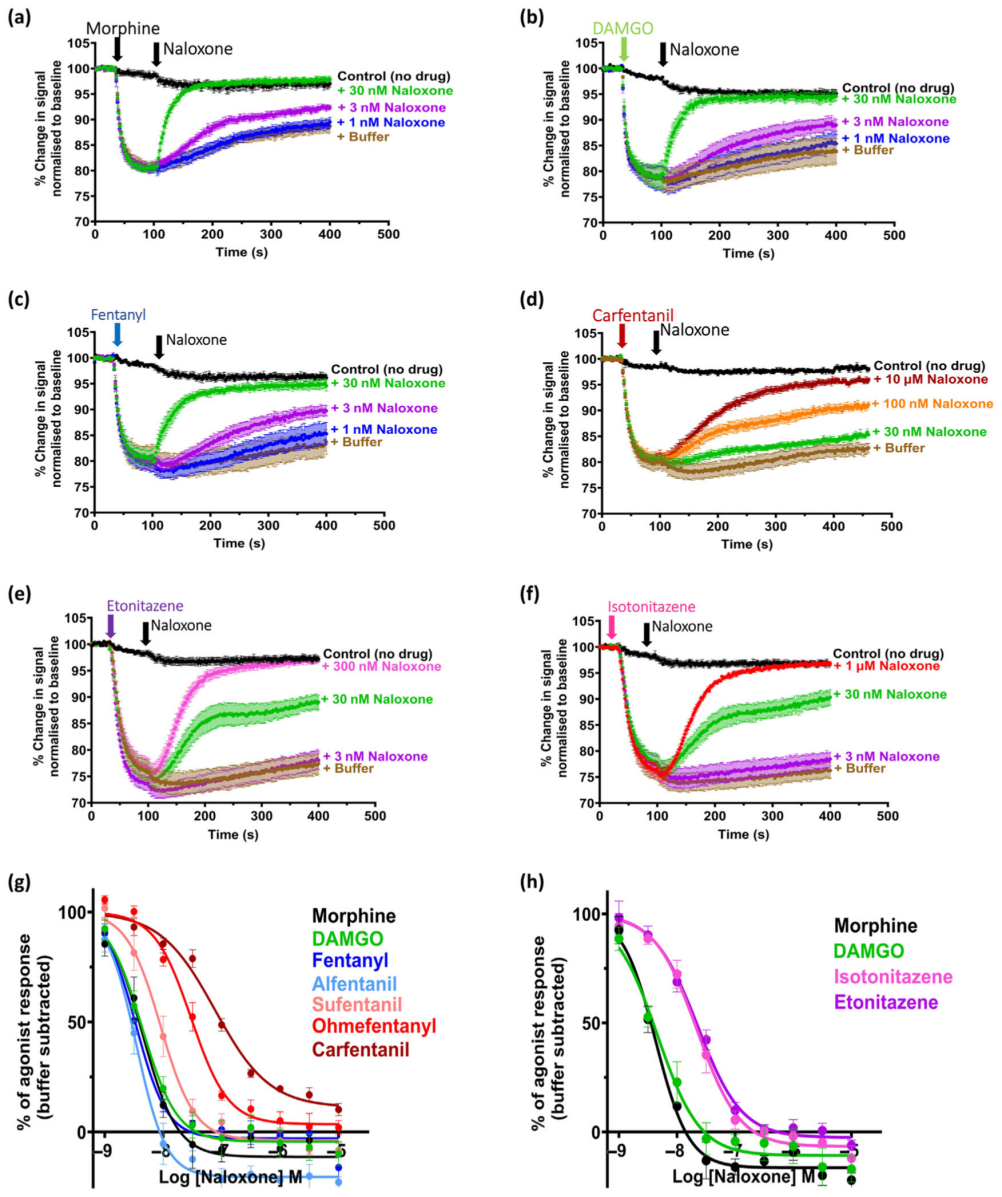
Concentration–response relationships for naloxone reversal of opioid-induced hyperpolarisation of *μ*-opioid receptor-expressing AtT20 cells. (a–f) Pooled experimental data showing the change in membrane potential dye fluorescence signal produced by the EC_75_ concentration of each of six opioid agonists—morphine, DAMGO, fentanyl, carfentanil, etonitazene and isotonitazene—and the subsequent reversal by addition of three concentrations of naloxone. Traces represent the mean ± SEM of five individual experiments for each drug. The black line ‘Control (no drug)’ represents no agonist and no naloxone addition, only equivalent volume of vehicle injections. (g and h) Log concentration–response curves for the reversal by naloxone of each opioid agonist obtained in experiments similar to those shown in Figure 2a–d. Data for DAMGO, fentanyl, alfentanil, sufentanil, ohmefentanyl and carfentanil shown in Figure 2g were obtained in a different set of experiments from those for etonitazene and isotonitazene shown in Figure 2h. To facilitate comparison of all opioid agonists tested, morphine and DAMGO were included in both sets of experiments. Data shown are means ± SEM, *n* = 5 for each drug. Log concentration–response curves were fitted using nonlinear regression with the top constrained to 100%. Calculated values of naloxone pIC_50_ against each agonist are given in Table 1.

In the emergency treatment of human opioid overdose, the antagonist naloxone is administered after the response to the agonist has developed. Therefore, we first sought to mimic this situation in our in vitro experiments by administering each opioid agonist, allowing the response to reach steady state, and then administering naloxone (see Figure 2a–f). Each agonist was added at its EC_75_ concentration, and the ability of a range of naloxone concentrations to reverse the agonist response was determined. The order of potency of naloxone to reverse the opioid agonists was morphine = DAMGO = fentanyl = alfentanil > sufentanil > etonitazene = isotonitazene > ohmefentanyl >> carfentanil (Figure 2g,h; see Table 1 for pIC_50_ values for naloxone).

#### 3.2.2 Competitive antagonism

**Figure 3:**
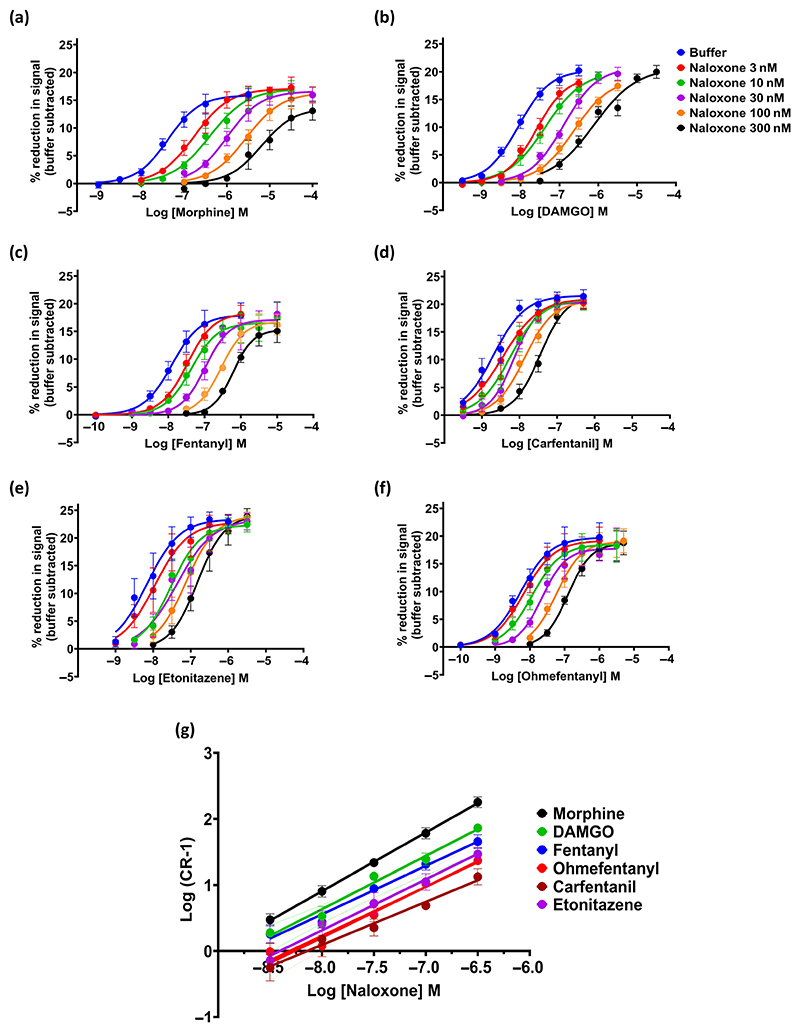
Schild analysis of the antagonism by naloxone of opioid-induced hyperpolarisation of *μ*opioid receptor-expressing AtT20 cells. For the Schild analysis, cells were exposed to naloxone at various concentrations for 30 min prior to addition of a range of concentrations of each opioid agonist. (a–f) Log concentration–response curves for six opioid agonists—morphine, DAMGO, fentanyl, carfentanil, ohmefentanyl and etonitazene—in the absence and presence of naloxone. Each data point represents the mean ± SEM of *n* = 5 observations. Log concentration–response curves were fitted using nonlinear regression with the bottom constrained to zero. (g) Schild plots of log (concentration ratio – 1) against log naloxone concentration for each of the opioid agonists tested. Data shown are means ± SEM, *n* = 5 for each drug. The data were fitted using simple linear regression. Calculated values of naloxone *pA*_2_ are given in Table 1.

Estimation of the pA_2_ and thus the equilibrium dissociation constant (K_D_) of a competitive antagonist requires that binding of the antagonist to the receptor is at equilibrium before the addition of the agonist to compete with the antagonist for binding. In theory, for competitive antagonism at the same receptor, the antagonist pA_2_ should be independent of the agonist as the antagonist binds to the receptor when it is unoccupied by the agonist (Kenakin, 1982). We therefore exposed μ-opioid receptor-expressing AtT20 cells to increasing concentrations of naloxone for 30 min at 37°C prior to determining the log concentration–response relationship of each agonist and used these data to measure the concentration ratio of the agonist (Figure 3). Naloxone (3–300 nM) produced increasing parallel shifts to the right of the concentration–response curve of each agonist with no decrease in maximum response. However, the degree of rightward shift produced by naloxone was not the same for each agonist (Figure 3a–f), and subsequent Schild analysis revealed that the naloxone pA_2_ was not the same for each agonist (Figure 3g and Table 1). For DAMGO, morphine and fentanyl, the pA_2_ values for naloxone (Table 1) were similar to the *K*_*i*_ value of 1.3 nM reported from radioligand binding studies on human μ-opioid receptors (Toll et al., 1998). However, for the other agonists, the naloxone pA_2_ was greater indicating that they were less sensitive to naloxone antagonism. The rank order of naloxone sensitivity was DAMGO = morphine = fentanyl > etonitazene > ohmefentanyl > carfentanil. Whilst the slopes of the Schild plots for DAMGO and morphine were 0.8 and 0.9, respectively, they were statistically different from unity as determined by the extra-sum-of-squares *F* test. For the other agonists, the slope was even lower, with carfentanil displaying the lowest slope (Table 1). This may be indicative of the interaction between naloxone and the fentanyl and nitazene agonists not being truly competitive in nature (see Section 4) or of the agonists and naloxone acting on more than one subtype of opioid receptor. Therefore, we have not converted the observed *pA*_2_ values to *K*_D_ values for the fentanyl and nitazenes analogues (Kenakin, 1982).

To exclude the possibility that the opioid agonists were activating more than one type of opioid receptor in the AtT20 cells, we examined whether *δ, κ* or NOP opioid receptors were endogenously expressed in the parent cell line, AtT20FlpInWT, in which the μ-opioid receptor had subsequently been recombinantly expressed. A commercial transcriptome analysis performed by Macrogen© had previously indicated that the AtT20FlpInWT cells did not endogenously express *δ* or *κ* opioid receptors and only endogenously expressed NOP opioid receptors at a very low level (the full transcriptome analysis data for AtT20FlpInWT cells have been made publicly available by Prof M. Connor, Macquarie University, Australia at 10.25949/21529404.v1). We attempted to confirm the absence of these opioid receptors by examining whether AtT20FlpInWT cells responded to opioid agonists selective for *δ, κ* or NOP opioid receptors. When we exposed AtT20FlpInWT cells (i.e. the parent cell line not recombinantly expressing the μ-opioid receptor) to SNC80 (1 μM), U69593 (10 μM) or nociceptin (1 μM), highly selective agonists at δ, *κ* and NOP opioid receptors, respectively, there was no decrease in fluorescence in cells loaded with membrane potential dye. This is consistent with these cells not endogenously expressing significant amounts of *δ, κ* or NOP opioid receptors that couple to GIRK channels. In addition, we sought to exclude the possibility that the high concentrations of carfentanil or etonitazene used to overcome antagonism by naloxone (Figure 3) might have off target effects at non-opioid receptors in AtT20 cells, so reducing the effectiveness of naloxone. When we exposed AtT20FlpInWT cells to carfentanil (100 nM) or etonitazene (300 nM), there was no change in fluorescence in cells loaded with membrane potential dye, indicating that, even at the high concentrations, carfentanil and etonitazene were devoid of off target actions that affected membrane potential.

### 3.3 Off rate of agonist binding

**Figure 4:**
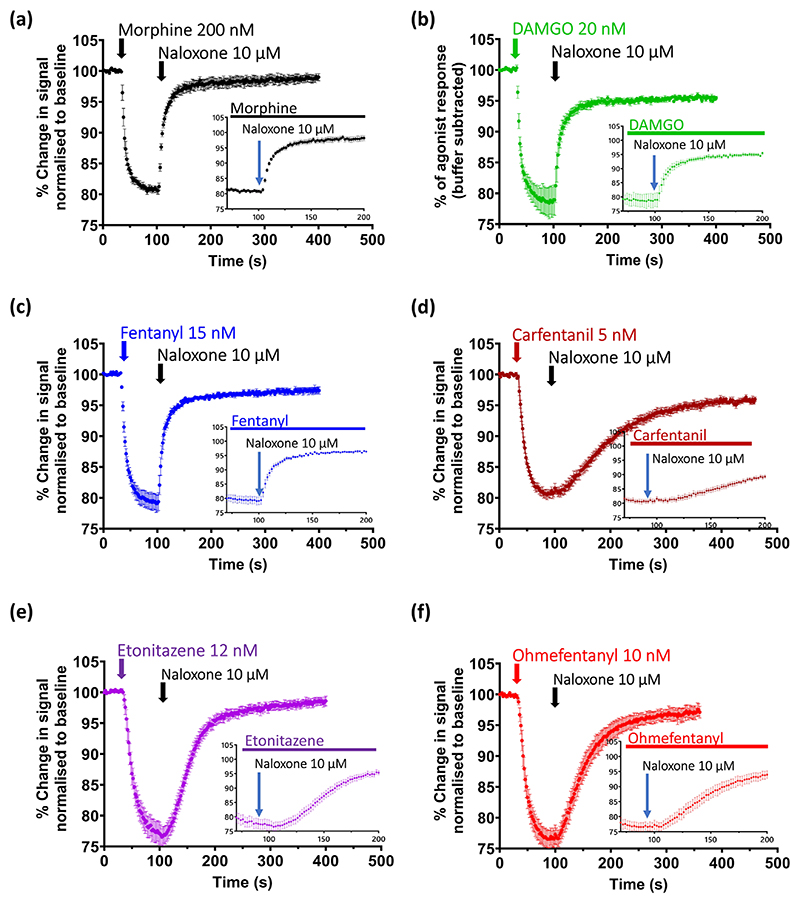
Rate of dissociation of opioid agonists from the *μ*-opioid receptor in AtT20 cells. (a–f) Pooled experimental data for each opioid agonist showing the change in membrane potential dye fluorescence signal produced by its EC_75_ concentration and subsequent reversal over time by the addition of a high concentration of naloxone (10 μM). Traces represent the mean ± SEM of five individual experiments for each drug. The inserts show, on an expanded timescale, the responses to naloxone to illustrate the delay to onset of response decay observed with carfentanil, ohmefentanyl and etonitazene but not with morphine, DAMGO and fentanyl. The calculated values for the *t*_1/2_ of apparent rate of dissociation and the apparent rate constant of dissociation of each are given in Table 1.

To examine whether the reduced sensitivity to reversal by naloxone of some fentanyls and nitazenes (see 3.2.1 above) might be because of slow agonist dissociation from the receptor, we measured the apparent rate of agonist dissociation by allowing the response to the EC_75_ concentration of each agonist to reach steady state before applying a receptor supersaturating concentration of naloxone (10 μM) (Figure 4). The decay phase of the agonist response in the presence of naloxone was fitted to a single exponential curve and the apparent *t*_1/2_ of dissociation was determined. For fentanyl, alfentanil, morphine, and DAMGO, the apparent rate of agonist dissociation was similar to the response time of our assay procedure, as the values of apparent *t*_1/2_ of dissociation obtained were similar to the estimated equilibrium time for mixing following drug injection into the medium bathing the cells in the wells of the plate reader (see Section 2). Therefore, the values for apparent *t*_1/2_ of dissociation given in Table 1 for these agonists are an upper limit rather than precise values. However, given that the other agonists examined gave longer apparent *t*_1/2_ of dissociation values, we can conclude that the rate of dissociation from the μ-opioid receptor for the agonists tested was: fentanyl, alfentanil, DAMGO and morphine > sufentanil > etonitazene = isotonitazene ≥ ohmefentanyl >> carfentanil (Table 1). Although in Figure 4d the response to carfentanil was not completely reversed by naloxone 10 μM over the 5 min of naloxone exposure, we subsequently performed a separate series of experiments where naloxone was applied for longer (11 min) and it completely reversed the response to carfentanil (Figure S1). In contrast to agonists showing rapid dissociation (e.g. morphine and fentanyl), for agonists exhibiting slow dissociation (e.g. etonitazene and carfentanil), there was a delay from addition of naloxone to the first observable decrease in the amplitude of the agonist response (see inserts in Figure 4a–f and Table 1).

**Figure 5:**
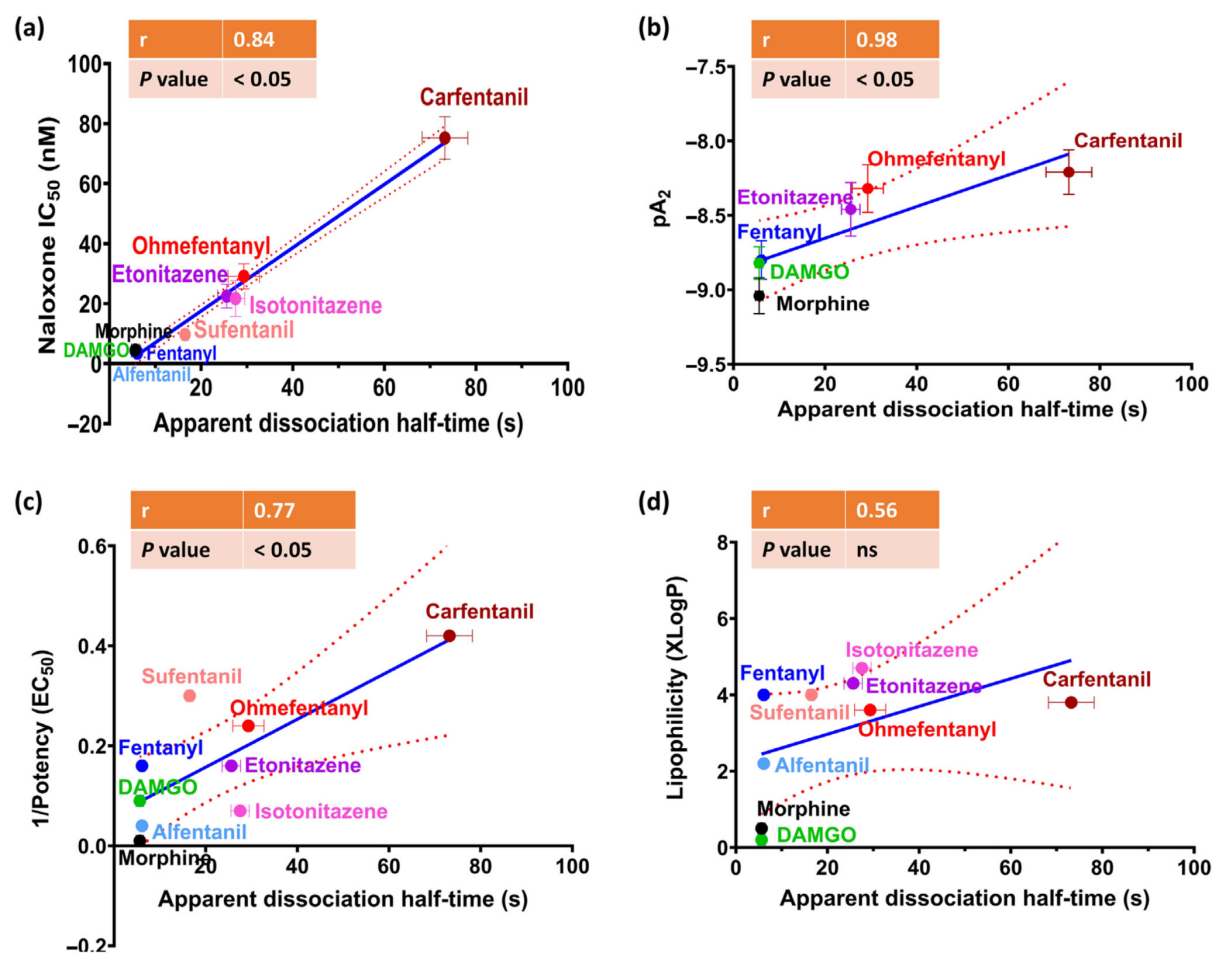
Correlation of apparent agonist dissociation time from the *μ* opioid receptor with susceptibility to antagonism by naloxone, agonist potency and lipid solubility. The graphs show the correlation between apparent dissociation half time (*t*_1/2_) with sensitivity to naloxone reversal(a), with the *pA*_2_ for naloxone reversal (b), with agonist potency (calculated as reciprocal of EC_50_) (c) and with agonist lipid solubility (XLogP values obtained from PubChem) (d). Data are shown as the mean ± SEM values for each agonist as given in Table 1. The r value and degree of significance obtained by linear regression analysis are indicated on each graph; the dotted lines denote the 95% confidence intervals for the solid regression line.

Figure 5a shows a graph of the correlation between the apparent rate of agonist dissociation from the μ-opioid receptor and the sensitivity to reversal by naloxone. For the nine agonists studied, there was a strong correlation between the apparent *t*_1/2_ of dissociation and reversal by naloxone. Similarly, there was a strong correlation between the apparent *t*_1/2_ of agonist dissociation and the *pA*_2_ for antagonism following prior exposure to naloxone (Figure 5b). There was a weaker correlation between the apparent *t*_1/2_ of agonist dissociation from the receptor and agonist potency and no observable correlation with lipophilicity (Figure 5c,d).

### 3.4 Opioid agonist signalling bias

**Figure 6:**
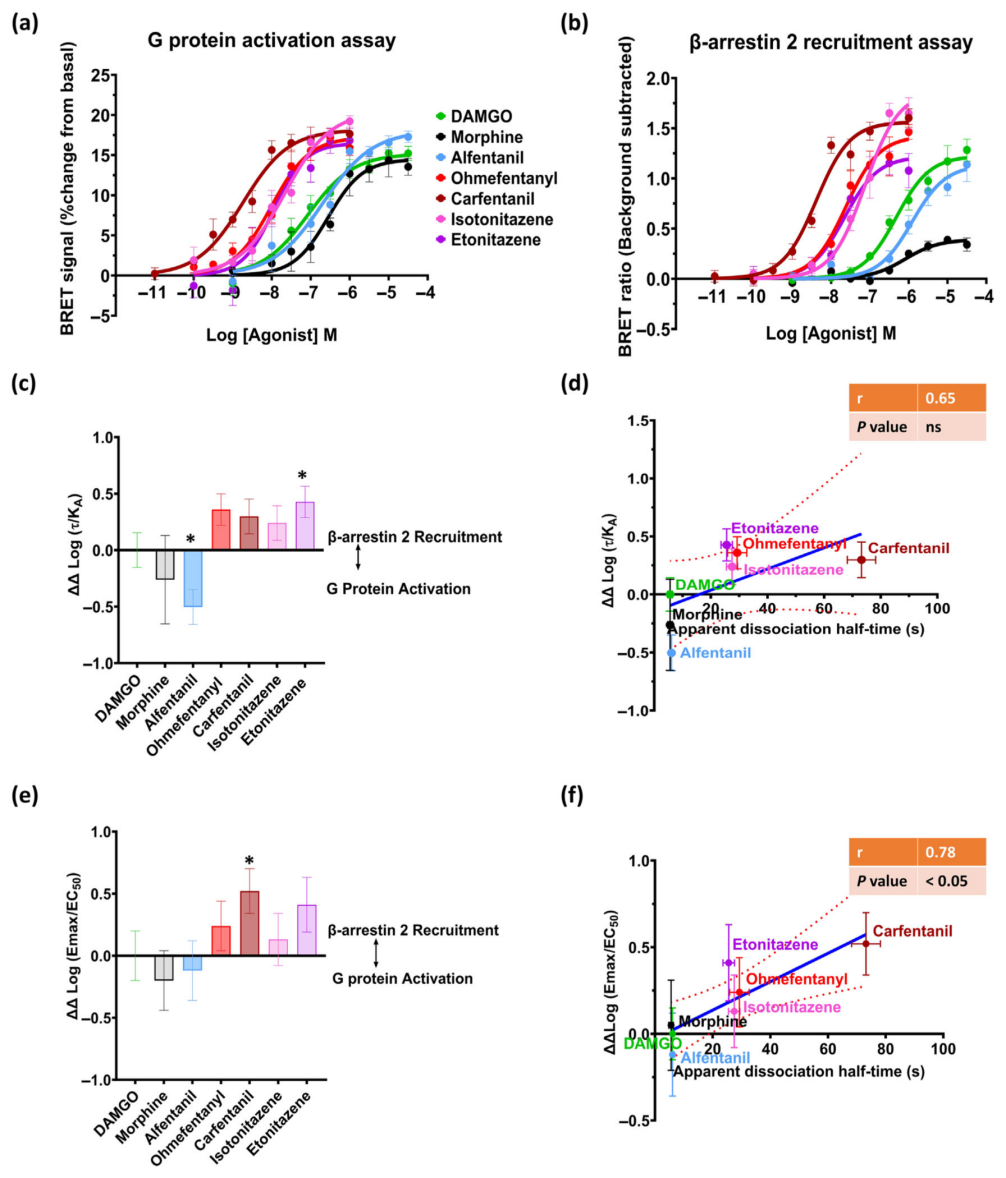
Estimation of opioid agonist bias between G protein activation and β-arrestin 2 translocation at the *μ*-opioid receptor. (a and b) Log concentration–response curves for opioid agonist-induced Gi protein activation and β-arrestin 2 translocation in HEK293T cells expressing the *μ* opioid receptor measured by BRET. Data are expressed as the mean ± SEM, *n* = *5* for each drug; G protein activation is expressed as a percent change from basal; β-arrestin 2 recruitment is expressed as raw BRET ratio with the background subtracted. Log concentration-response curves were fitted using nonlinear regression with the Hill slope constrained to 1 and the bottom of the curve constrained to zero. (c and e) From the data in panels (a) and (b), signalling bias was quantified in two ways: using (ΔΔ Log [*τ*/*K*_A_]) and ΔΔ log (*E*_max_/EC_50_) (see Section 2 for details). * denotes statistical significance (P<0.05) from 0 (for DAMGO) using a one-sample two-tailed *t*-test. (d and f) The graphs show the correlation between apparent dissociation half time (*t*_1/2_) and (ΔΔ Log (*τ*/*K*_A_)) or ΔΔ Log (*E*_max_/EC_50_) values for the agonists studied. The values shown are the mean ± SEM values for each agonist. The *r* value and degree of significance obtained by linear regression analysis are indicated on the correlation graphs; the dotted lines denote the 95% confidence intervals for the solid regression line.

We have shown previously on three separate occasions that fentanyl is unbiased (McPherson et al., 2010; Rivero et al., 2012; Ramos-Gonzalez et al., 2023). However, carfentanil shows moderate bias for β-arrestin translocation over G protein activation (Ramos-Gonzalez et al., 2023). We therefore sought to examine whether slow dissociation from the μ-opioid receptor was associated with bias for β-arrestin 2 translocation over G protein activation. We compared the ability of DAMGO and morphine (as reference ligands), alfentanil (rapid-dissociating agonist), ohmefentanyl, carfentanil, isotonitazene and etonitazene (slow-dissociating agonists) to activate G protein or recruit β-arrestin 2 in HEK293T cells expressing the μ-opioid receptor (Figure 6).

The rank order of potency for G protein activation was: carfentanil > ohmefentanyl > etonitazene > isotonitazene > DAMGO > alfentanil > morphine (Table 2). Similarly, in the β-arrestin 2 translocation assay, the rank order of potency was: carfentanil > ohmefentanyl = etonitazene > isotonitazene > DAMGO > morphine > alfentanil, with morphine exhibiting weak partial agonist activity in this assay (Table 2). Whilst there was some variability in *E*_max_ values for the agonists in each assay, all three fentanyls and DAMGO signalled with a similar *E*_max_ for G protein activation (Figure 6a). Isotonitazene, however, exhibited a significantly higher *E*_max_ compared to DAMGO. In the β-arrestin 2 translocation assay, the *E*_max_ values of isotonitazene and carfentanil were significantly higher than that of DAMGO.

Bias was quantified using both the operational model (Log (τ/*K*_A_) and Log (*E*_max_/EC_50_)). For each method, two statistical tests were performed: one-way ANOVA and *t*-test (see statistical analysis section). These two statistical tests revealed different outcomes regarding the bias profile of some agonists. For example, carfentanil was β-arrestin 2 biased when bias was quantified using the Log (*E*_max_/EC_50_) method and a one-sample two-tailed *t*-test, whereas etonitazene but not carfentanil was β-arrestin 2 biased when bias was quantified using the operational model method and a one-sample two-tailed *t*-test.

Despite the contrasting statistical findings, correlation analyses of the bias values and apparent dissociation half-times revealed a significant correlation between the apparent dissociation half-time of the agonist and ΔΔ log (*E*_max_/EC_50_) (Figure 6f). In contrast, the correlation was weaker and not significant between the apparent dissociation half-time of the agonist and ΔΔ Log (*τ*/*K*_A_) (Figure 6).

### 3.5 Arrestin binding and agonist off rate

**Figure 7:**
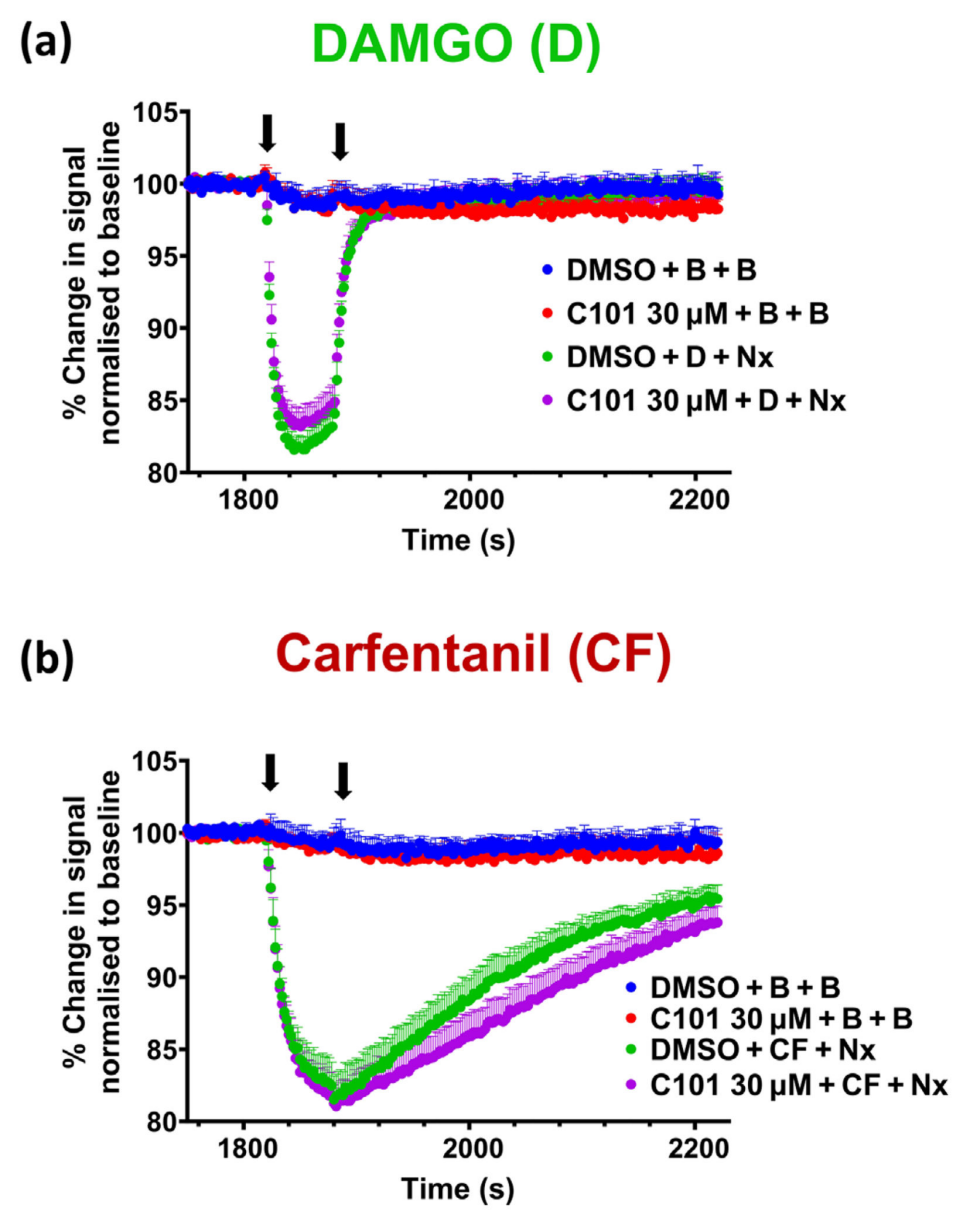
Effect of compound 101, a G protein receptor kinase (GRK) inhibitor, on the rate of dissociation of agonists from the *μ*-opioid receptor in AtT20 cells. Cells were pretreated with 30 μM Compound 101 (C101) for 30 min before the administration of the EC_75_ concentration of (a) DAMGO or (b) carfentanil. Naloxone (10 μM) was administered 60 s post-agonist. Traces represent the mean ± SEM of five individual experiments for each agonist. C101, Compound 101; CF, carfentanil; D, DAMGO; Nx, 10 μM naloxone; B, buffer; DMSO, 0.01%.

One possibility is that opioid agonist-induced G protein-coupled receptor kinase (GRK) phosphorylation and arrestin binding induces a conformational change in the orthosteric pocket of the μ-opioid receptor that decreases agonist dissociation rate. Such an effect would be most likely to affect the dissociation of those agonists that showed a tendency towards β-arrestin bias. We therefore examined whether the GRK2 and GRK3 inhibitor, Compound 101 (Cmpd101), which reduces agonist-induced μ-opioid receptor phosphorylation and subsequent β-arrestin binding (Lowe et al., 2015), altered the apparent agonist dissociation rate from the μ-opioid receptor. DAMGO and carfentanil were examined as the former is a neutral agonist with rapid dissociation and the latter is β-arrestin biased with slow dissociation. Pretreatment of AtT20 cells expressing the μ-opioid receptor with Cmpd101 (3–30 μM) failed to increase the apparent rate of dissociation of either carfentanil or DAMGO (Figure 7). Indeed, Cmpd101 slightly slowed the apparent rate of dissociation of carfentanil, but the effect, whilst consistent, was not marked, and there was no slowing of the apparent rate of dissociation of DAMGO. This suggests that neither GRK phosphorylation and subsequent β-arrestin binding to the μ receptor traps opioid agonists in the orthosteric binding pocket of the receptor.

## 4 Discussion

When compared to morphine as the standard opioid agonist, the rank order of potency of the fentanyls and nitazenes to produce membrane hyperpolarisation in vitro was largely as expected (Tables 1 and 3). These relative potency values agree with those reported previously using a variety of in vitro assays involving G protein activation (Åstrand et al., 2020; Emmerson et al., 1996; Faouzi et al., 2022; Glatfelter et al., 2023; Malcolm et al., 2023; McPherson et al., 2010; Ramos-Gonzalez et al., 2023; Toll et al., 1998; Vandeputte et al., 2021; Vandeputte et al., 2023).

Across a number of in vivo studies of antinociception, the relative potency of fentanyls and nitazenes compared to morphine is much higher than that observed using in vitro assays (Table 3). Furthermore, the change in relative potency between in vitro and in vivo assays was not consistent across the range of fentanyls tested. For fentanyl, the difference was around 20-fold but for carfentanil it was 350-fold. Fentanyl and nitazene analogues are highly lipophilic, but this cannot fully explain the disparity between their in vitro and in vivo relative potencies as carfentanil and fentanyl are of similar lipophilicity (XlogP values of 4.0 and 3.8, respectively), but the enhanced relative potency of carfentanil in vivo was much greater. Fentanyl, alfentanil and morphine are substrates for P-glycoprotein-mediated extrusion from the brain (Dagenais et al., 2004; Kalvass et al., 2007; F. Martins et al., 2023; Yu et al., 2018). However it seems unlikely that the in vitro–in vivo relative potency disparity between fentanyl/alfentanil and carfentanil/ohmefentanyl/isotonitazene/etonitazene could be attributed to P-glycoprotein, excluding fentanyl and alfentanil from the brain more effectively, as pharmacological blockade or genetic deletion of P-glycoprotein only increases the brain levels of fentanyl and alfentanil by threefold (Kalvass et al., 2007; Yu et al., 2018). The mechanisms responsible for the enhanced potency of some fentanyls and nitazenes in vivo, as well as the much greater in vivo potency of carfentanil relative to fentanyl remain unexplained.

A key finding of this study was that the apparent rate of drug dissociation from the μ-opioid receptor varied significantly between the opioid agonists, alfentanil and fentanyl dissociating rapidly and carfentanil very slowly. It is unlikely that the measured apparent rate of dissociation of the opioid agonists was influenced by receptor desensitisation or internalisation as the submaximal concentrations of each agonist applied induced little apparent desensitisation over the 10 min of agonist exposure. The apparent rate of dissociation of carfentanil observed was much faster than that reported by Mann et al. (2022)—(73 and 2800 s, respectively). This may be because of differences in experimental conditions between the studies. Our study was conducted on intact cells in the presence of physiologically relevant concentrations of Na^+^ and guanine nucleotide, whereas Mann et al. used membrane homogenates and a zero Na^+^ and guanine nucleotide buffer, which would enhance agonist affinity and slow the rate of agonist dissociation (Mohell & Nedergaard, 1985). An early study also measured carfentanil dissociation from the μ-opioid receptor in membrane homogenates but in the presence of Na^+^ and guanine nucleotide and reported the off-rate to be 10-fold faster than Mann et al. (Titeler et al., 1989).

The manner in which opioid agonists interact with residues in the orthosteric binding pocket of the μ-opioid receptor might explain their different dissociation rates. Whilst a recent cryoEM study (Zhuang et al., 2022) demonstrated that in the orthosteric binding pocket of the μ-opioid receptor fentanyl interacts with amino acid residues in transmembrane domains II, III, V, VI and VII, docking experiments in the same study suggested that carfentanil, through its methoxycarbonyl group, forms additional interactions with I298^6.61^, W320^7.35^ and I324^7.39^. We have also observed such interactions following molecular dynamics analysis of carfentanil binding to the active structure of the μ-opioid receptor (Ramos-Gonzalez et al., 2023), whilst lofentanil, which contains a methoxycarbonyl group like carfentanil, also interacts with the above three residues in the orthosteric pocket (Qu et al., 2022). Such additional interactions could decrease the dissociation rate of carfentanil relative to fentanyl and explain the higher affinity of carfentanil for the μ-opioid receptor.

Apart from the orthosteric pocket, it is possible that interactions of carfentanil and similar ligands with other receptor regions, such as a potential pathway between the transmembrane domains into the membrane (Sutcliffe et al., 2022), or a potential vestibule site (Dror et al., 2011), could also hinder the dissociation of carfentanil. Regarding the latter, the dissociation of LSD (lysergide) from the 5HT_2B_ receptor is greatly slowed by the ECL2 of this receptor acting as a ‘lid’ (Wacker et al., 2017), whilst binding to residues in the vestibule region specifically hinders the dissociation of the antagonist tiotropium from the muscarinic M_3_ receptor (Kistemaker et al., 2019); further studies will be required to see if such mechanisms operate for carfentanil and other slowly dissociating ligands from the μ-opioid receptor. Potential interactions with the receptor on the way to and from the orthosteric pocket could also enhance the process of agonist rebinding, such that the ‘on rate’ of agonist binding also becomes an important factor contributing to agonist residence time at the receptor (Lane et al., 2017). We were unable to measure the on rate of agonist binding and μ-opioid receptor activation, as the on rate of the response to all the agonists studied was faster than the response time of our assay system. Additionally, in conditions of limited diffusion, such as neuronal synapses, the on rate may play a role in prolonging the apparent lifetime of agonist binding through the process of rebinding (Vauquelin & Charlton, 2010).

We have previously reported that carfentanil exhibits bias towards β-arrestin recruitment over G protein activation at the μ-opioid receptor (Ramos-Gonzalez et al., 2023), whereas fentanyl is unbiased (McPherson et al., 2010; Rivero et al., 2012; Ramos-Gonzalez et al., 2023). In the present study, we observed that carfentanil and etonitazene exhibited a tendency for β-arrestin bias. Fentanyl and etonitazene have previously been shown to induce phosphorylation of the μ-opioid receptor by GRK 2 and 3, thus facilitating β-arrestin recruitment (for review and references, see Duarte & Devi, 2020; see also Underwood et al., 2024). However, the GRK 2/3 inhibitor C101 (Lowe et al., 2015) did not enhance the apparent dissociation rate of carfentanil, rather, it slightly decreased it. This finding suggests that agonist-induced GRK phosphorylation and subsequent β-arrestin recruitment does not in some way trap potentially β-arrestin-biased opioid agonists in the orthosteric pocket of the μ-opioid receptor, slowing their dissociation. The β-arrestin bias of LSD at the 5-HT_2B_ receptor appears to be related to its long residency time in the orthosteric pocket of this receptor (Wacker et al., 2017). Slow agonist dissociation kinetics for dopamine receptor agonists have also been associated with ligand bias (Klein Herenbrink et al., 2016).

Another important finding of this study was that slowly dissociating agonists such as carfentanil were more resistant to antagonism by naloxone. There have been numerous reports of more naloxone, in the form of multiple or higher doses, being required to reverse overdoses involving fentanyls and nitazenes compared with heroin overdoses (Amaducci et al., 2023; Irvine et al., 2022; Moe et al., 2020). What may be occurring with the fentanyls could in fact be ‘over’ overdose, that is, far too high a dose of a fentanyl has been unintentionally administered, thus requiring more naloxone for reversal (Rzasa Lynn & Galinkin, 2018). We and others have shown in mice that higher doses of naloxone were required to reverse non-fatal fentanyl respiratory depression than to reverse morphine (Elder et al., 2023; Hill et al., 2020), although a recent study in rats reported equal sensitivity of fentanyl, carfentanil and heroin to naloxone (Hiranita et al., 2024).

The prerequisites for concluding that the interaction between agonists and an antagonist is competitive are (i) a parallel shift to the right of each agonist concentration response curve in the presence of increasing concentrations of the antagonist, (ii) no decrease in the maximum response of the agonists in the presence of the antagonist, (iii) the slope of the Schild plot should be unity and (iv) the *pA*_2_ value of the antagonist should be the same for all agonists acting at the same receptor. In the present in vitro study, higher concentrations of naloxone were required to antagonise some fentanyls (sufentanil, ohmefentanyl and carfentanil) and nitazenes (isotonitazene and etonitazene) irrespective of whether the naloxone was applied before or after the agonist. Surprisingly, given our in vivo mouse respiration data (Hill et al., 2020), fentanyl showed similar reversal by naloxone as morphine in vitro. For all of the agonists studied, antagonism was indeed surmountable, that is, there was a parallel shift to the right of the concentration–response curve in the presence of increasing concentrations of naloxone with no decrease in maximum agonist response. However, the slopes of the Schild plots for fentanyl, ohmefentanyl, carfentanil, etonitazene and isotonitazene were lower than unity, and the intercepts on the *X*-axis (*pA*_2_ values) increased in parallel with the reduction in slope. The results of the Schild analysis are incompatible with competitive agonist–antagonist interaction at the orthosteric binding site of a single receptor type. Given that the AtT20 cells we used in our assay only expressed μ-opioid receptors, we therefore conclude that naloxone antagonism of some fentanyls (fentanyl, ohmefentanyl and carfentanil) and etonitazene at the μ-opioid receptor is pseudo-competitive in nature, that is, some, but not all, of the prerequisites for competitive antagonism were met. The strong correlation between the apparent rate of agonist dissociation from the receptor and susceptibility to naloxone antagonism indicates that slow agonist dissociation impairs naloxone antagonism, rendering it pseudo-competitive. In a similar way, the in vivo actions of the slowly dissociating CB_1_ cannabinoid receptor agonist, HU-210, are less susceptible to antagonism by rimonabant than other faster-dissociating agonists at the CB_1_ receptor (Hruba & McMahon, 2014). Other potential mechanisms that might contribute to reduced sensitivity of some μ-opioid receptor agonists to naloxone include differential binding of some agonists to orthosteric and vestibule sites (discussed earlier) on the receptor, the latter reducing access of the antagonist to the orthosteric site. Alternatively, some fentanyls and nitazenes may act both at the orthosteric site as agonists and at an allosteric site to reduce the affinity of naloxone binding (Livingston & Traynor, 2018; O’Brien et al., 2024). Also, highly lipophilic agonists may be able to access the orthosteric pocket of the μ-opioid receptor both by the aqueous route and through the transmembrane helices of the receptor whilst naloxone uses only the aqueous route (Kelly et al., 2023; Sutcliffe et al., 2022). These potential mechanisms would result in nonequilibrium conditions and reduce antagonist sensitivity (Kenakin, 1982). It will be important to extend the naloxone antagonism experiments described in this paper to lipophilic antagonists (e.g. diprenorphine), hydrophilic antagonists (e.g. CTOP) and the irreversible antagonist beta-funaltrexamine.

## 5 Conclusions

Our data confirm the view that, in overdoses involving fentanyls and nitazenes, higher doses of the antidote naloxone may be required for reversal than those normally used to reverse heroin overdose. With slowly dissociating μ-opioid receptor agonists, antagonism by naloxone becomes pseudo-competitive. This indicates that the kinetics of the agonist should be factored in when evaluating the nature of antagonism.

## Supplementary Material

Supporting Information

Additional supporting information can be found online in the Supporting Information section at the end of this article.

Supplementary Figure

## Figures and Tables

**Table 1:** Data obtained from agonist concentration–response, naloxone reversal and agonist dissociation kinetic experiments.

	(i) Agonist concentration-response data				(ii) Naloxone antagonism				(iii) Agonist dissociation kinetics		
Agonist	pEC_50_^¶^	Maximum response (% morphine 1 μM) ^¶^	Hill slope^¶^		Naloxone *p*IC_50_ ± SEM^§^	Naloxone pA_2_ ± SEM^¥^	Slope of Schild plot^¥^		Apparent rate of dissociation (*t*_1/2_ s)^ǂ a^	Apparent rate constant of dissociation (*k*_off_ s ^−1^)	Naloxone time to onset of reversal (s)
Fentanyl	−8.19 ± 0.06*	124.7 ± 4.0*	1.3 ± 0.2		−8.46 ± 0.05	−8.80 ± 0.13	0.73 ± 0.07		6.1 ±0.5^b^	0.113	4
Alfentanil	−7.64 ± 0.04*	105.9 ± 6.7	1.9 ± 0.3		−8.40 ± 0.07	-	-		6.1 ±0.5^b^	0.113	2
Morphine	−7.14 ± 0.06 −6.94 ± 0.06	107.3 ± 2.0 111.7 ± 3.4	1.0 ± 0.1 1.1 ±0.1		−8.37 ± 0.05	−9.04 ± 0.12	0.89 ± 0.06		5.6 ± 0.4^b^	0.124	2
DAMGO	−7.98 ± 0.04* −8.12 ± 0.11*	117.0 ± 2.5 120.5 ± 3.5	1.6 ± 0.3 1.5 ± 0.2		−8.31 ± 0.07	−8.82 ± 0.11	0.81 ± 0.06		5.5 ± 0.2^b^	0.126	2
Sufentanil	−8.48 ± 0.05*	120.7 ± 6.1	1.5 ± 0.2		−8.01 ± 0.08*	-	-		16.5 ± 0.7*	0.042	6
Isotonitazene	−7.86 ± 0.07*	133.2 ± 8.3*	1.7 ± 0.4		−7.66 ± 0.11*	-	-		27.5 ± 2.0*	0.025	12
Etonitazene	−8.19 ± 0.15*	121.5 ± 4.0	2.2 ± 0.3		−7.65 ± 0.08*	−8.46 ± 0.18*	0.77 ± 0.08		25.6 ± 1.3*	0.027	14
Ohmefentanyl	−8.38 ± 0.10*	130.3 ± 2.7*	1.3 ± 0.2		−7.54 ± 0.06*	−8.32 ± 0.16*	0.79 ± 0.07		29.3 ± 3.4*	0.024	10
Carfentanil	−8.61 ± 0.09*	133.0 ± 2.7*	1.0 ± 0.1		−7.12 ± 0.04*	−8.21 ± 0.15*	0.65 ± 0.12		73.2 ± 4.2*	0.009	20

(i) Agonist concentration-response data. ^¶^EC_50_s, maximum responses and Hill slopes for each agonist were determined from the concentration-response curves in Figure 1. Values represent the mean ± SEM of five individual experiments. For DAMGO and morphine, the concentration-response values are reported twice as the experiments were conducted as two different sets at different times. The closeness of the values for these two drugs indicates good reproducibility. (ii) Naloxone antagonism. ^§^Naloxone IC_50_s were calculated from naloxone reversal of the EC75 concentration of each agonist as shown in Figure 2. ^¥^pA2 and slope values were calculated from the Schild plots shown in Figure 3. (iii) Agonist dissociation kinetics. ^ǂ^*t*_1/2_ values were obtained by fitting a single exponential to the time course of the reversal of the EC_75_ concentration of each agonist by naloxone (10 μM) as shown in Figure 3 and described in Section 2.5. ^a^In the data from individual experiments used to calculate mean ± SEM values for *t*_1/2_ for each agonist, the goodness of fit was R ≥ 0.8. ^b^The ability to measure rapid agonist displacement by naloxone is limited by the rate of drug mixing in the well after injection into the fluid bathing the cells which was estimated to be approximately 5.8 s. Thus, the off rate of these drugs is likely to be faster than the *t*_1/2_ measured by this method. *Indicates statistically significant difference (*p* <0.05) compared to morphine using one-way ANOVA with Dunnett’s post hoc test.

**Table 2:** Opioid agonist potency in G_i_ protein activation and β-arrestin 2 translocation BRET assays in HEK293T cells expressing the μ-opioid receptor.

	G_i_ protein assay^¶^			β-arrestin 2 assay^¶^	
Agonist	Agonist pEC_50_ ± SEM	Agonist *E*_max_ % change from basal		Agonist pEC_50_ ± SEM	Agonist *E*_max_ (raw BRET signal, basal subtracted)
Carfentanil	−8.58 ± 0.17*	17.5 ± 0.96		−8.38 ± 0.08*	1.6 ± 0.08*
Ohmefentanyl	−8.09 ± 0.12*	16.6 ± 0.9		−7.55 ± 0.12*	1.5 ± 0.07
Etonitazene	−7.90 ± 0.18*	16.6 ± 1.4		−7.60 ± 0.04*	1.3 ± 0.1
Isotonitazene	−7.67 ± 0.18*	20.5 ± 0.8*		−7.07 ± 0.16*	1.99 ± 0.2*
DAMGO	−6.82 ± 0.06	15.5 ± 0.9		−6.12 ± 0.14	1.2 ± 0.1
	−6.99 ± 0.19	14.6 ± 0.6		−6.33 ± 0.05	1.2 ± 0.1
Alfentanil	−6.53 ± 0.22	17.5 ± 0.23		−5.84 ± 0.16	1.2 ± 0.1
Morphine	−6.32 ± 0.04	14.0 ± 0.7		−6.17 ± 0.24	0.34 ± 0.02*
	−6.38 ± 0.21	14.6 ± 1.0		−6.01 ± 0.12	0.39 ± 0.04*

^¶^EC_50_s and maximum responses for each agonist were determined from the concentration−response curves in Figure 6. Values represent the mean ± SEM of 5 different experiments performed in duplicate.

*Indicates a significant difference (p < 0.05) compared to DAMGO using one-way ANOVA with Dunnett’s multiple comparisons test. Two sets of experiments were conducted at different times. Each data set was compared to an internal DAMGO control, hence there are two DAMGO data sets (morphine was also repeated and its two data sets are included for information).

**Table 3:** Comparison of relative potencies of fentanyls and nitazenes to morphine in an in vitro assay and in vivo antinociception assays.

	Potency relative to morphine	
Agonists	In vitro membrane potential assay (this study)	In vivo assays of antinociception (average of multiple studies)
Morphine	1	1
Carfentanil	29×	~10,200
Sufentanil	22 ×	~3500×
Ohmefentanyl	17×	~6300×
Etonitazene	11×	~1400×
Isotonitazene	8×	~800×
Fentanyl	11×	~200×
Alfentanil	3×	~80×

Data for relative potencies in rodent thermal antinociception assays (hot plate and tail flick) were compiled and averaged from values previously reported for fentanyls by Jin et al. (1981), Kalvass et al. (2007), Mather (1983), Niemegeers et al. (1976), Wynn et al. (1986), and Van Daele et al. (1976) and for nitazenes by De Luca et al. (2022), Glatfelter et al. (2023), Hunger et al. (1960), Jacobson (1992), Vandeputte et al. (2023), and Walker and Young (2001).

## Data Availability

The data that support the findings of this study are available from the corresponding author upon reasonable request.

## What is already known

- ‘Fentanyls’ and ‘nitazenes’ are potent agonists at the μ-opioid receptor.

## What does this study add

- Some fentanyls and nitazenes dissociate slowly and are less sensitive to naloxone antagonism

## What is the clinical significance

- More naloxone may be required to reverse overdoses involving fentanyls and nitazenes

## Abbreviations

BRET: Bioluminescence Resonance Energy Transfer
Cmpd101: Compound 101
Cryo-EM: cryogenic electron microscopy
DMEM: Dulbecco's modified Eagle’s medium
ECL: extracellular loop
GIRK: G protein-coupled inwardly rectifying potassium channel
GRK: G protein-coupled receptor kinase
HEK293T: human embryonic kidney 293
RlucII: *Renilla* luciferase II.
