# Supplementary material for: Slow dissociation kinetics of fentanyls and nitazenes correlates with reduced sensitivity to naloxone reversal at the μ-opioid receptor
Source: Br J Pharmacol. Author manuscript; Available in PMC 2025 Sep 15. (PMC7618119; doi:10.1111/bph.17376)
Supplement: Supplementary Figure [file EMS207948-supplement-Supplementary_Figure.pdf]

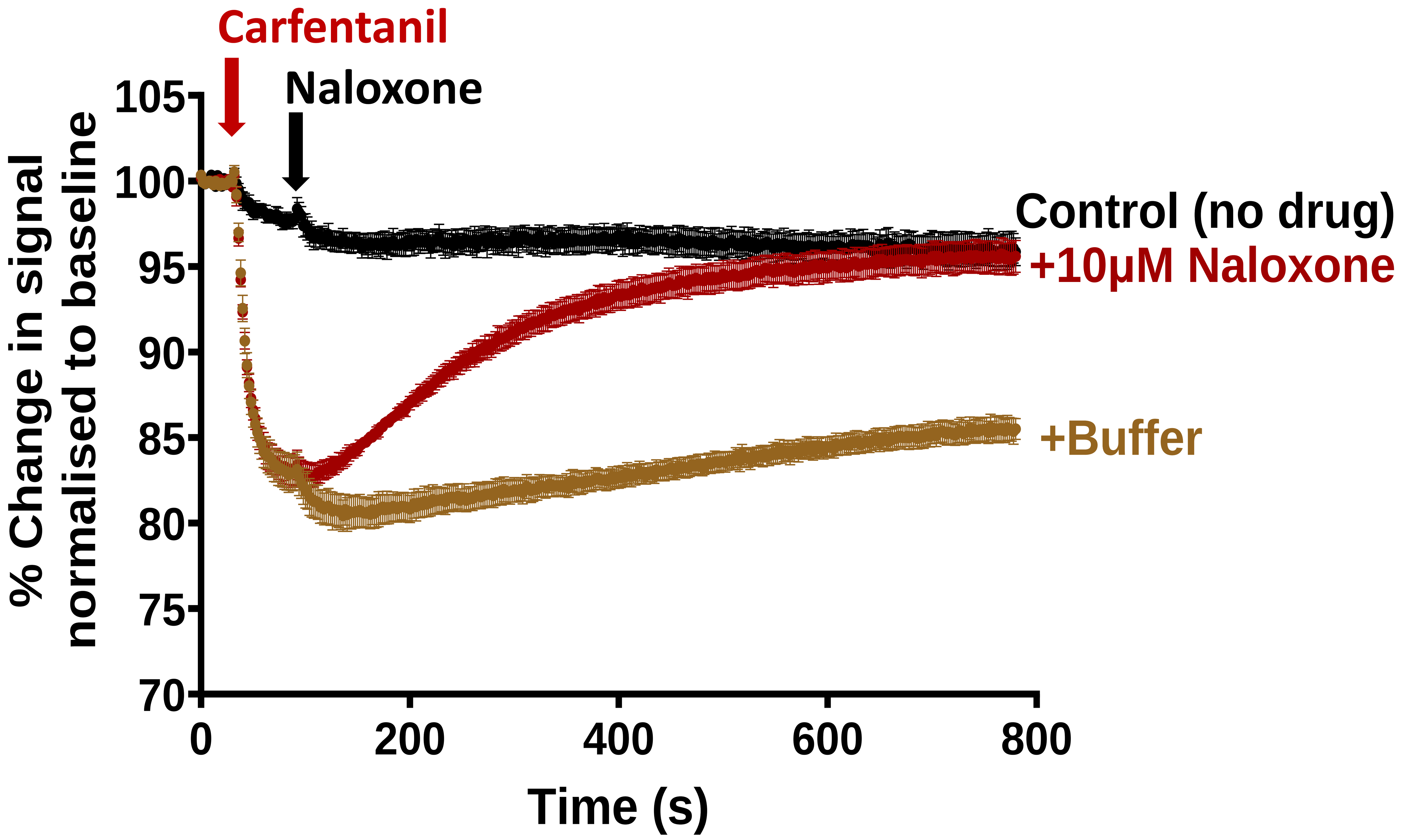

Supplementary Figure 1. Complete reversal by naloxone of the carfentanil-induced hyperpolarisation of μ opioid receptor-expressing AtT20 cells.

Pooled experimental data showing the change in membrane potential dye fluorescence signal produced by the EC_{75} concentration of carfentanil and subsequent reversal over time by addition of a high concentration of naloxone (10 mM). Traces represent the mean ± SEM of 5 individual experiments.
